# Microscale Gene Expression Analysis of Tumor-Associated Macrophages

**DOI:** 10.1038/s41598-018-20820-4

**Published:** 2018-02-05

**Authors:** Kuldeep S. Attri, Kamiya Mehla, Surendra K. Shukla, Pankaj K. Singh

**Affiliations:** 1 The Eppley Institute for Research in Cancer and Allied Diseases, University of Nebraska Medical Center, Omaha, Nebraska USA; 2 Department of Biochemistry and Molecular biology, University of Nebraska Medical Center, Omaha, Nebraska USA; 3 Department of Pathology and Microbiology, University of Nebraska Medical Center, Omaha, Nebraska USA; 4 Department of Genetics Cell Biology and Anatomy, University of Nebraska Medical Center, Omaha, Nebraska USA

## Abstract

Macrophages, apart from being the key effector cells of the innate immune system, also play critical roles during the development and progression of various complex diseases, including cancer. Tumor-associated macrophages, infiltrate tumors during different stages of cancer progression to regulate motility, invasion, and intravasation to metastatic sites. Macrophages can exist in different polarization states associated with unique function in tumors. Since tumor-associated macrophages constitute a very small proportion of tumor cells, analysis of gene expression pattern using normal extraction buffer-based methods remains a challenging task. Therefore, it is imperative to develop low-throughput strategies to investigate transcriptional regulations from a small number of immune cells. Here, we describe an efficient, sensitive, and cost-effective approach for gene expression analysis of a small number of fluorescence-activated sorted tumor-associated macrophages. Our analyses from the different number of stable, primary, and sorted macrophages suggest 5,000 cells is an optimal number for performing quantitative, real-time PCR analysis of multiple genes. Our studies could detect expression of macrophage-specific genes from cultured primary macrophages, and FACS-sorted macrophages from different biological tissues without introducing biases in comparative gene expression ratios. In conclusion, our kit-based method for quantitative gene expression analysis from a small number of cells found in biological tissues will provide an opportunity to study cell-specific, transcriptional changes.

## Introduction

Macrophages are terminally differentiated phagocytic cells of the innate immune system, differentiated from monocytes that are derived from hematopoietic stem cell precursors^1^. Found as circulating and tissue-resident cells, macrophages can polarize into classically or alternatively activated subtypes. M1 macrophages are classically activated, pro-inflammatory cells involved in evoking the inflammatory response and pathogen clearance^2^. M2 macrophages are alternatively activated, anti-inflammatory cells involved mainly in wound healing and regeneration^3,4^. Apart from these roles, macrophages known as tumor-associated macrophages (TAMs) also play an important role in cancer progression^5^.

Macrophages are particularly abundant in tumor sites and constitute a major fraction of non-malignant cell populations in the tumor microenvironment^6^. Macrophages are also found in different stages of cancer, in various cancer types, in varying abundance^7^. Multiple correlations have also been established between modulation of the tumor microenvironment and macrophage polarization status^8,9^. Recent data suggests opposing roles for M1 and M2 macrophages in modulating tumor biology^10,11^. While M2 macrophages are pro-tumoral in primary and metastatic sites, M1 macrophages are anti-tumoral in action^12^. M2 macrophages stimulate angiogenesis and enhance tumor invasion and intravasation properties to regulate metastatic spread. Conversely, M1 macrophages mediate immunosuppressive function by preventing activated natural killer and T-cells from tumor cell killing^13^. Various subpopulations of macrophages are said to regulate different aspects of tumor biology, making them an interesting subject of study.

The transcriptomic studies in monocytes and polarized macrophages suggest remarkable differences in the gene expression of subtypes^14^. Microarray and next generation high-throughput techniques such as RNA-Seq are commonly employed to investigate global gene expression changes; however, qualitative expression changes in a small number of genes is checked by quantitative real-time polymerase chain reaction (qRT-PCR)^15^. The qRT-PCR technique is very commonly used to study gene expression from a large number of cells; however, achieving optimal RNA yields for qRT-PCR analysis from a small number of cells has always been challenging^16,17^. With recent advances in technology, gene expression analysis from single cells is also possible, although it involves the introduction of amplification steps that can introduce biases, and requires expertize to perform complex high-throughput data analysis^18^. Apart from these limitations, there are also very limited studies that have described methods to achieve quantitative gene expression from a small number of cells^19^. To overcome the limitation of pooling samples for the study of gene expression, there is an urgent need to develop methods and pipelines to enable qRT-PCR analysis from a small number of isolated cells.

As noted above, macrophages are known to play crucial effector roles in various diseases of different tissue origins^20^. Macrophages can also respond to different microenvironmental cues that trigger their differentiation to multiple subpopulations with distinct transcriptional profiles^21^. Since these different subpopulations can exist in varying proportions in different tissues in both healthy and disease states, it is imperative to understand transcriptional rewiring that occurs in these cells and is critical for regulating tissue biology. Although numerous gene expression studies have been conducted on tissue and/or tumor macrophages, hardly any studies have been conducted from a small number of input TAMs to understand transcriptional changes *in vivo*^22,23^. Therefore, we have developed a fast, highly reproducible and sensitive method called the “Microscale gene expression analysis method,” which enables us to study transcriptional regulations from as few as 5,000 fluorescence-activated sorted macrophages.

## Results

### Determination of the Minimum Cell Number for Gene Expression Analysis in U937 Cells

**Figure 1:**
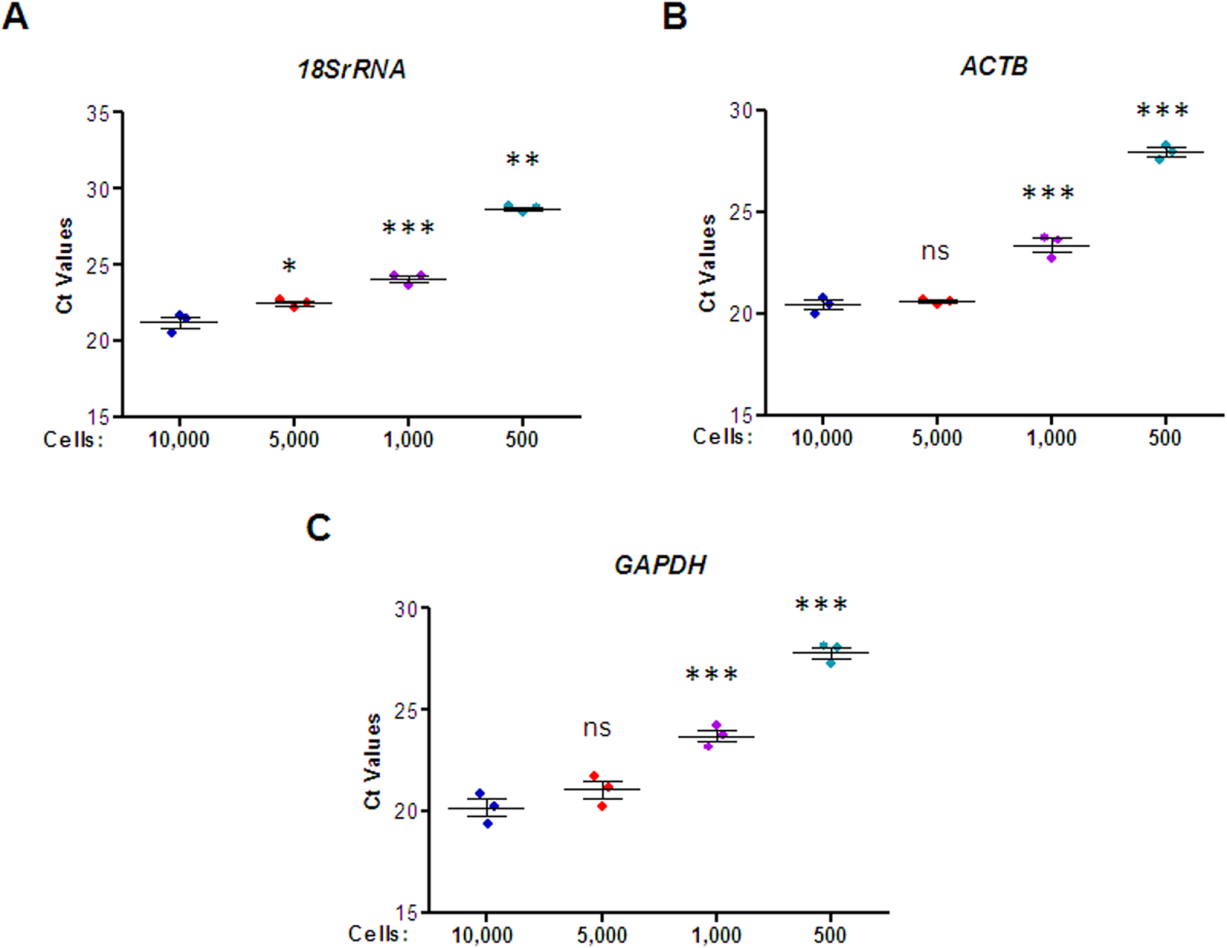
Comparative expression analysis of housekeeping genes from the different number of U937 cells. The graphs show Ct values of (**A**) *18SrRNA*, (**B**) *ACTB* and (**C**) *GAPDH* from a different number of U937 cells. The cDNA was probed at 1:10 dilution. The data is represented as mean ± SEM from n = 3. The significant differences in mean Ct values of samples with different cell numbers was compared to 10,000 cells by one-way ANOVA. ***Denotes p-value < 0.001, **Denotes p-value < 0.01, *Denotes p-value < 0.05 and ns stands for non-significant.

To develop a method for gene expression analysis from a small number of macrophages, especially TAMs, we decided to work with a varied number of input U937 monocytes, which are the stable cell precursors of macrophages. We initially isolated RNA from 10,000, 5,000, 1,000 and 500 cells using PicoPure RNA isolation kit, which works on a column-based purification method. Because the RNA isolated by this method is in very low quantity and cannot be quantified on nanodrop, we chose to synthesize cDNA by using SuperScipt VILO Mastermix. From the synthesized cDNA, we then performed qRT-PCR analysis at the dilution of 1:10 to check for the expression of three housekeeping genes (*18SrRNA*, *ACTB*, and *GAPDH*). It was very interesting to find that the transcripts for all three housekeeping genes were detected from different numbers of cells (Fig. 1). A Ct value difference of 1 was seen between 10,000 and 5,000 cells and a difference of 2–3 Ct value was observed between 10,000 and 5,000 cells. However, a huge difference in Ct values was observed between 10,000 and 500 cells for all three housekeeping genes. The results obtained through our method showed detectable gene expression from a surprisingly small number of cells, even as low as 500.

Further, we probed the cDNA at a lower dilution of 1:20 for all three housekeeping genes to optimize cDNA dilution for gene expression studies. Ct values for all three housekeeping genes were also detected in U937 cells at different number of input cells. Also at the cDNA dilution of 1:10, a consistent difference of 1 Ct value was observed between 10,000 and 5,000 cells for all three housekeeping genes (Figure S1). We then tested the presence of genomic DNA contamination in RNA samples by performing qRT-PCR analysis from (+) RT and (−) RT samples. In (−) RT samples, reverse transcriptase enzyme was not added. We could detect a Ct difference of more than 9 Ct values between (+) RT and (−) RT reactions from 10,000, 5,000 and 1,000 cells for the *ACTB* gene, suggesting no possible genomic DNA contamination in the RNA samples (Figure S1A). A similar trend was also noted for the other two housekeeping genes (Figures S1B and S1C). However, the Ct value difference between (+) RT and (−) RT reactions for 500 cells was less than 9 Ct values, suggesting probable DNA contamination in the samples. A Ct value difference of 10 suggests no possible genomic DNA contamination because the relative abundance of target transcripts between the two reactions is very high. Our experiments also suggest 10,000 to 1,000 as an ideal cell number for U937 cells to probe for gene expression at a cDNA dilution of 1:20.

### Optimization and Reproducibility of Microscale Gene Expression Analysis Method

**Figure 2:**
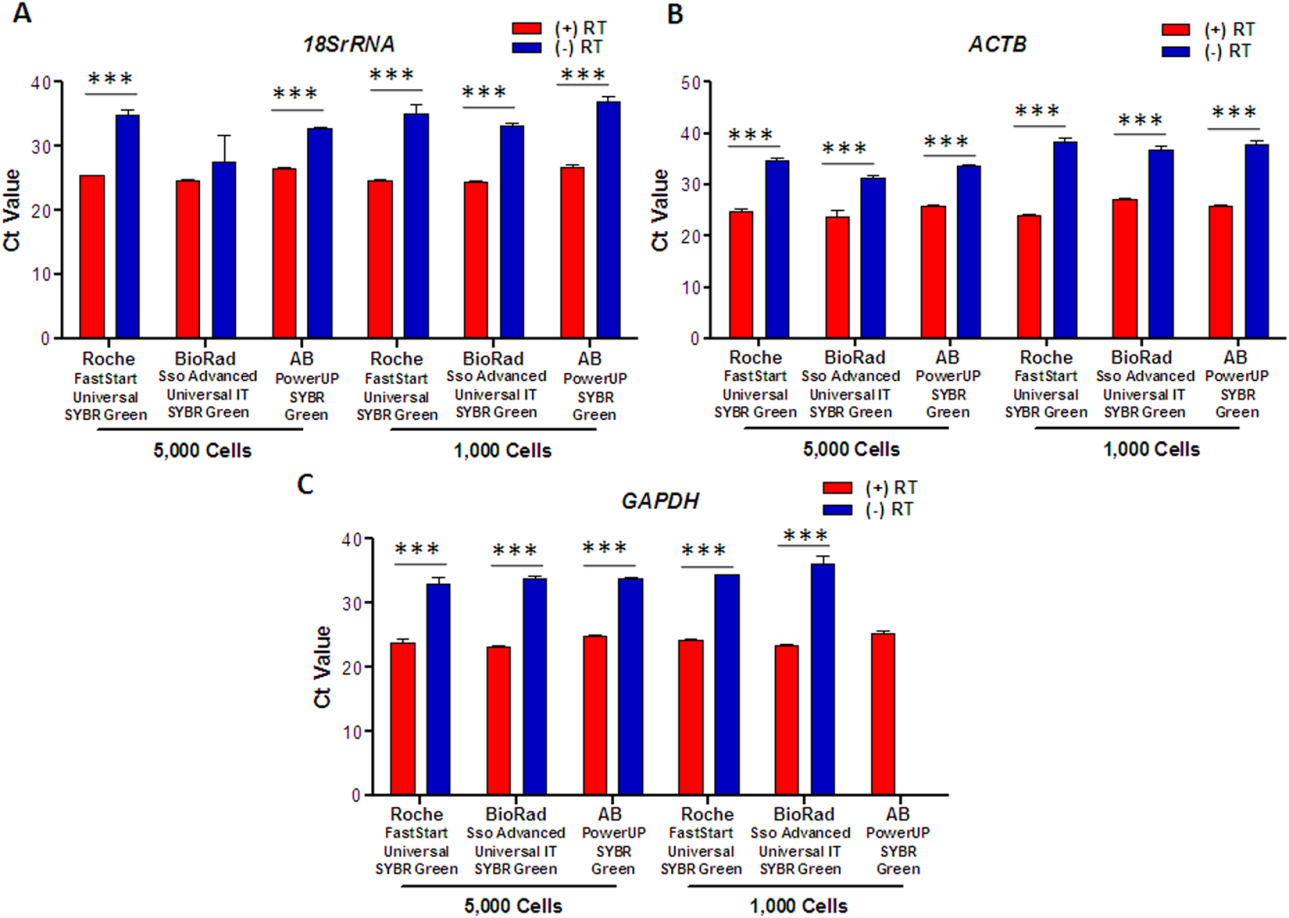
Optimization of SYBR Green qPCR mix for gene expression detection in a low number of U937 cells. The plots represent Ct values of (**A**) *18SrRNA*, (**B**) *ACTB* and (**C**) *GAPDH* in (+) RT and (−) RT reactions synthesized using Roche FastStart Universal SYBR Green, BioRad Sso Advanced Universal IT SYBR Green, and AB PowerUP SYBR Green MasterMixes. The cDNA synthesized from 5,000 and 1,000 U937 cells was probed at 1:20 dilution of cDNA. The data is represented as mean ± SEM from n = 3. The comparison of significant differences in mean Ct values of (+) RT and (−) RT groups for an individual reagent was compared using one-way ANOVA. ***Denotes p-value < 0.001, **Denotes p-value < 0.01, *Denotes p-value < 0.05 and ns stands for non-significant.

There are three main steps in the gene expression analysis method: RNA isolation procedure, cDNA synthesis, and qPCR reactions with SYBR Green dye reagent. In earlier results, the first two steps were optimized for RNA isolation by PicoPure RNA isolation kit and cDNA synthesis by SuperScript VILO Mastermix. Next, we wanted to test if the use of different SYBR reagents could account for increased sensitivity and reproducibility across different experimental setups. For this test, we used three different SYBR dyes: Roche FastStart Universal SYBR Green, BioRad Sso Advanced Universal IT SYBR Green, and AB PowerUP SYBR Green to determine the most sensitive dye compatible for our method. Because the aim was to detect expression from a lower number of cells, we performed SYBR dye comparisons in an experiment conducted with 5,000 and 1,000 input cells, at a dilution of 1:20 for (+) RT and (−) RT-PCR reactions. The expression of the *18SrRNA* gene was found to be significantly consistent with the use of AB PowerUP SYBR Green dye, and showed minute variations between the experimental replicates (Fig. 2A). Next, we compared the differences between (+) RT and (−) RT-PCR reactions conducted with different SYBR Green dyes. The experiment performed with Roche FastStart Universal SYBR Green and AB PowerUP SYBR Green dye showed significant differences in Ct values of *18SrRNA*, *ACTB*, and *GAPDH* genes. The variation between the different experimental replicates was also significantly less compared to Bio-Rad SYBR dye (Fig. 2). From this point, we named our methodology, using the above-mentioned pipeline, “Microscale gene expression analysis method.” This method involves isolation of RNA by PicoPure RNA Isolation kit, followed by cDNA synthesis from isolated RNA by SuperScript VILO enzyme mix, and finally the qRT-PCR reaction with AB Hotstart SYBR dye.

We further tested our methodology for reproducibility in the sensitivity of gene detection. We performed the experiment in triplicate with the same amount of input cells from different experimental setups. The analysis of *18SrRNA*at a cDNA dilution of 1:20 (from 5,000 cells) using our Microscale gene expression analysis method found no significant variations in Ct values from the three biological replicates (Figure S2A). Further, analysis of *ACTB* and *GAPDH* genes also showed a similar trend, with no considerable variation observed among the three biological replicates and indicating high reproducibility in conducting the experiment (Figures S2B and S2C). However, the variation in Ct values of all three housekeeping genes from cDNA (1:20 dilution) of 1,000 U937 cells showed very high variation among biological replicates (Figure S2). The large variation observed in the experiment could have arisen because of inefficiency in the RNA isolation procedure using a small number of cells, or due to biases introduced during the cDNA amplification step from the isolated input RNA. Transcript detection at cDNA dilution of 1:20 conveniently allows probing for more than 100 genes. Thus, our methodology is well-suited to perform reproducible quantitative gene expression analysis of a large number of genes from a minimum of at least 5,000 U937 monocytic cells.

### Comparative Gene Expression Analysis from a Large and Small Number of Cells

**Figure 3:**
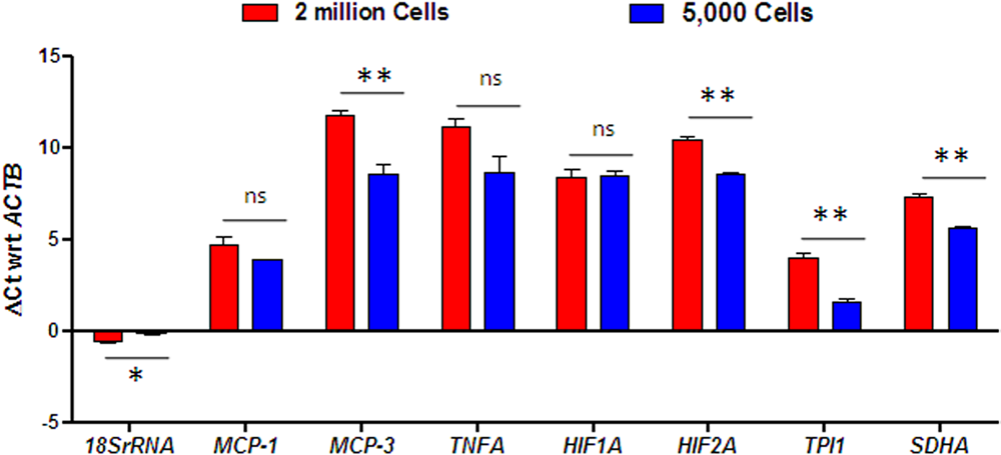
Comparative analysis of ΔCt in RNA isolated from U937 macrophages by TRIzol method and Microscale gene expression method. The bar plot represents ΔCt values of various genes with reference to the human *ACTB* gene in cDNA synthesized from RNA using two different methodologies for the high and low number of U937 cells. The data is represented as mean ± SEM from n = 3. The statistical significance between experimental groups was determined using unpaired two-tailed student’s t-test. ***Denotes p-value < 0.001, **Denotes p-value < 0.01, *Denotes p-value < 0.05 and ns stands for non-significant.

The transcripts of housekeeping genes such as *18SrRNA*, *ACTB*, and *GAPDH* are present in high abundance in the cells, and the transcript number remains more or less constant under different treatment conditions. Since the housekeeping genes are detected at low Ct values due to their high abundance, we wanted to test further if our method could also detect the presence of low abundant transcripts in the cell. We thus extended our methodology to detect expression of a panel of other target genes that includes a few key genes responsible for monocyte and macrophage migration (*MCP-1* and *MCP-3*), cytokines (*TNFA*), hypoxia-regulated genes (*HIF1A* and *HIF2A*), and metabolic genes (*TPI1* and *SDHA*). We performed qRT-PCR analysis on the RNA isolated from 5,000 U937 cells for detection of various target genes. The detectable expression of all seven genes was recorded at 1:20 dilution of cDNA using our method (Fig. 3), wherein the RNA isolated from a small number of cells is used to synthesize cDNA. We compared our methodology with the most commonly used TRIzol method for RNA isolation, followed by cDNA synthesis by Verso kit. We wanted to test whether there might be any bias introduced in Ct value ratios of different genes detected by both methodologies. We compared the Ct values of seven target genes and two housekeeping genes (*ACTB* and *GAPDH*) normalized to another housekeeping gene *18SrRNA*. The RNA isolated from 2 million cells by TRIzol method when tested for the seven target genes showed detectable Ct values for all genes tested. Our comparative analysis found the same trend in expression of all nine genes detected by our method with RNA isolated from 2 million cells by TRIzol method (Fig. 3). Apart from TPI-1, all genes showed similar levels of ΔCt values, and no significant variation was observed in ΔCt value for half of target genes. We concluded that our method can detect expression of low transcripts in the cell and does not introduce any significant biases in relative expression of target genes. These results confirm our method is effective to analyze gene expression from a small number of cells.

### Validation of Gene Expression Analysis Method in Murine Bone Marrow-derived Macrophages

**Figure 4:**
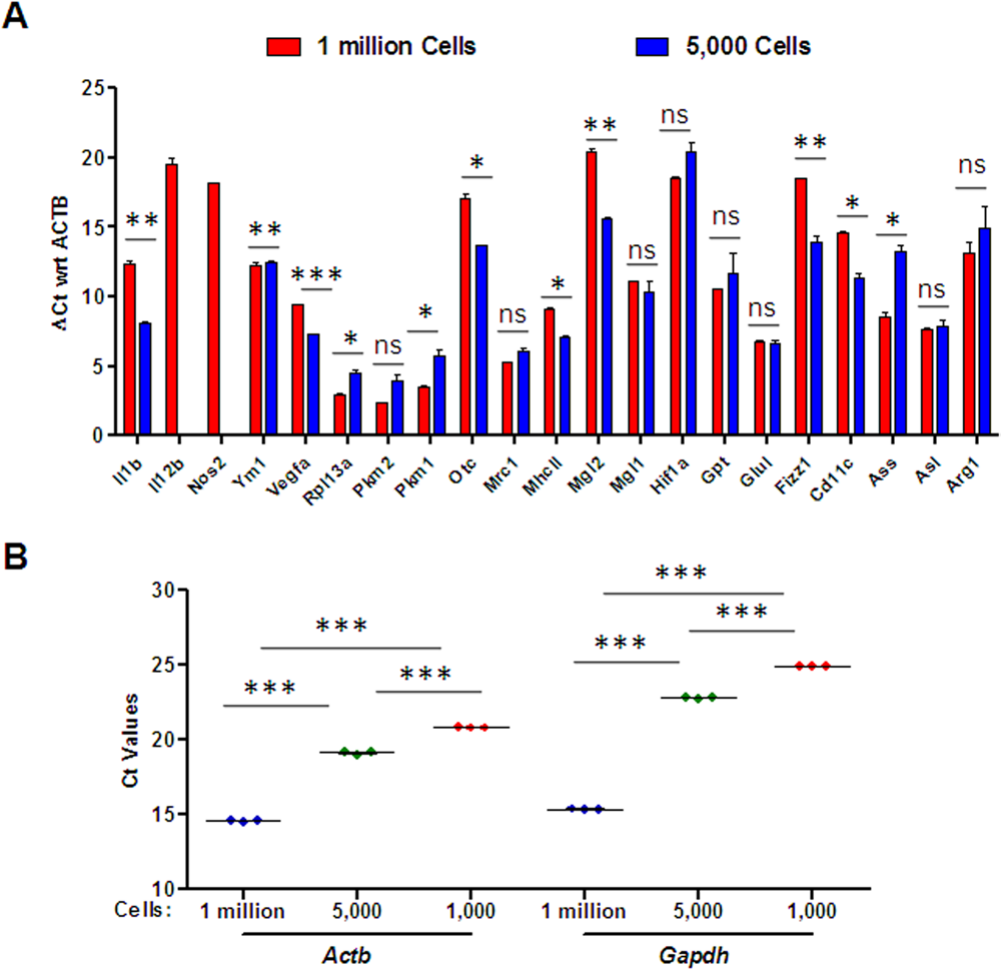
Comparative ΔCt analysis of macrophage-specific genes from a small number of murine bone marrow-derived macrophages. (**A**) The bar plot shows comparative expression profile of macrophage-specific genes from 1 million murine BMDMs isolated by TRIzol method and 5,000 murine BMDMs by Microscale gene expression method. The ΔCt values of various genes is derived with reference to the murine *Actb* gene, the candidate reference gene. The data is represented as mean ± SEM from n = 2. Statistical significance between experimental groups was determined using unpaired two-tailed student’s t-test. (**B**) The graph represents variability in expression of *Actb* and *Gapdh* gene from three different experiments. The RNA isolated from 1 million, 5,000, and 1,000 murine BMDMs was probed at the cDNA dilution of 1:20. The data is represented as mean ± SEM from n = 3. The significant difference in mean ∆Ct values of samples with different cell numbers was compared using one-way ANOVA. ***Denotes p-value < 0.001, **Denotes p-value < 0.01, *Denotes p-value < 0.05 and ns stands for non-significant.

To prove the adaptability of the proposed method to the different types of macrophages, we validated the experiments done earlier onU937 human cells in the primary murine bone marrow-derived macrophages (BMDMs). The BMDMs are primary cells cultured *ex-vivo* from mouse bone marrows, and we speculated that murine BMDMs are probably more similar to macrophages isolated from the different kinds of tissues. To replicate the results standardized with U937 cells, we isolated RNA from 5,000 murine BMDMs and analyzed expression of genes by our Microscale gene expression analysis method. Next, we constructed a panel of 21 macrophage-specific genes, including M1 and M2 subtype-specific genes. Macrophages can be polarized into either M1 or M2 macrophages which play different roles in cancer and other complex diseases^24^. The different subtypes of macrophages express subtype-specific markers like iNOS by M1 macrophages and Arg1 by M2 macrophages^25^. Ct values were detected for majority of the genes from 5,000 murine BMDMs, except for the *Il1b* and *Il12b* genes (Fig. 4A). Both of these are cytokine genes and expressed abundantly in M1 macrophages. In contrast, Ct values were detected from all genes when probed with cDNA synthesized from 1 million murine BMDMs by Trizol method, followed by cDNA synthesis by Verso kit. However, the expression of almost half the genes could not be detected by gene expression analysis done by our method from 1,000 cells, suggesting that 5,000 is the ideal low input cell number to study gene expression from *ex-vivo* cultured primary cells (data not shown). We then compared the relative abundance of several macrophage-specific genes upon normalization with the *Actb* gene, looking at between 5,000 and 1 million murine BMDM cells. Our analysis showed a similar trend in the regulation of all genes as depicted by almost equal ΔCt values of the target genes (Fig. 4A). ΔCt values between 1 million and 5,000 BMDMs were not statistically significant for approximately half of the target genes. Next, we tested the sensitivity of this method in primary murine BMDMs from 5,000 and 1,000 input cells in comparison to 1 million mBMDMs analyzed by the standard method. We found significant differences in Ct values of *Actb* and *Gapdh* genes in a different number of input cells. However, Ct value was only 5 unit higher in 5,000 cells compared to 1 million cells (Fig. 4B). Our results in conclusion suggest that our method is robust for performing gene expression analysis in a small number of primary macrophage cells.

### Standardization of Gene Expression Analysis Method for Fluorescence-activated Sorted Macrophages

**Figure 5:**
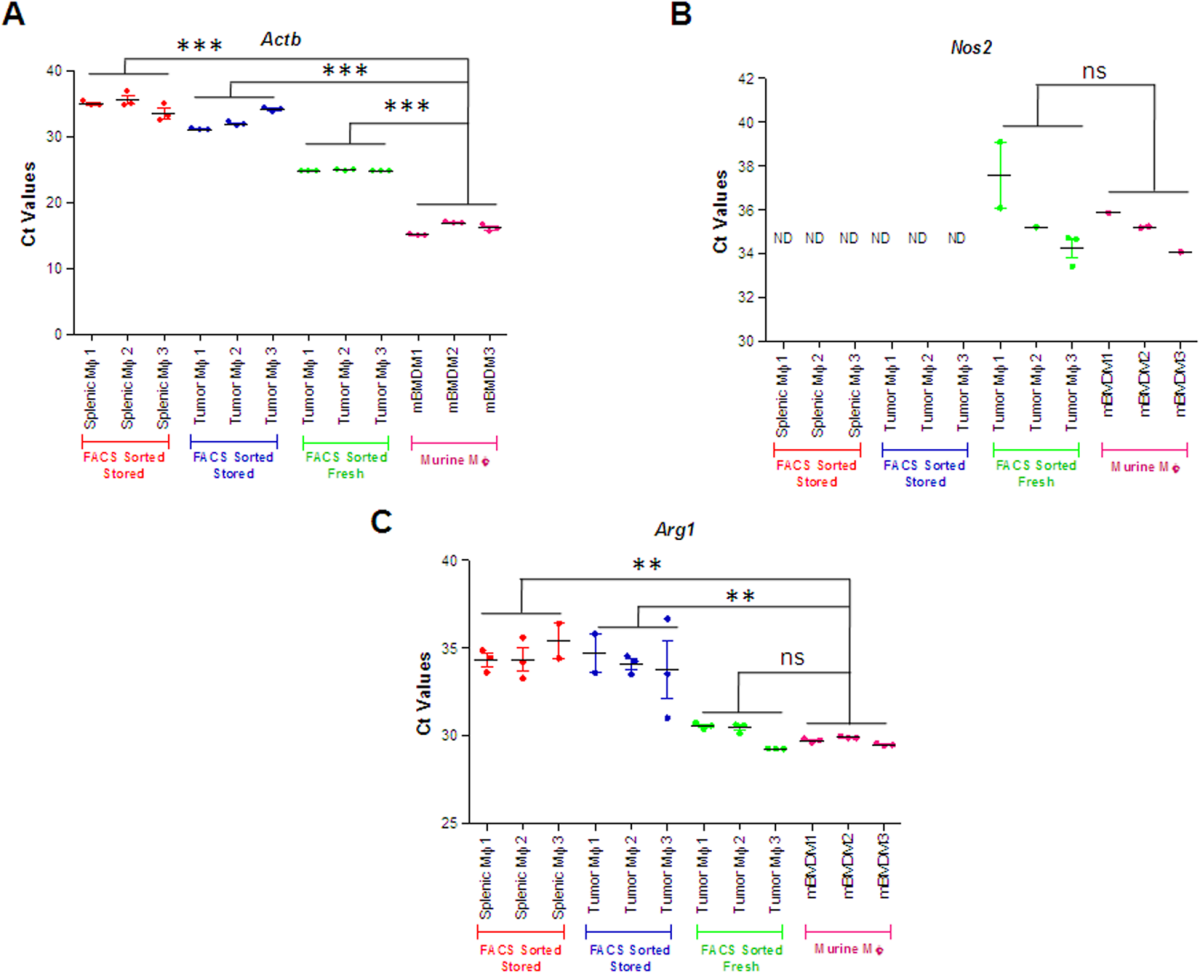
Gene expression analysis of macrophage markers in a small number of FACS-sorted macrophages. The plots represent relative expression of (**A**) *Actb*, (**B**) *Arg1*-M2 macrophage marker, and (**C**) *Nos2*-M1 macrophage marker in stored FACS-sorted splenic macrophages, stored FACS-sorted tumor macrophages, cultured murine BMDMs, and fresh FACS-sorted tumor macrophages by Microscale gene expression method. Three biological replicates were used for different macrophages. Each data point is represented as mean ± SEM from n = 3. The significant differences in mean Ct values of FACS sorted samples was compared to isolated murine macrophages by one-way ANOVA. ***Denotes p-value < 0.001, **Denotes p-value < 0.01, *Denotes p-value < 0.05 and ns stands for non-significant.

Macrophages are known to infiltrate tumors during the progression and metastasis of cancer^26^. The macrophage subtypes have differential relative abundance during the progression and metastasis of tumors. To test the gene expression from macrophages isolated from tumors, we orthotopically implanted KPC-k1 cells in the pancreas of 6–8-week-old female C57BL/6 albino mice. After tumor formation, tumors were excised to isolate macrophages by FACS sorting of pancreatic tumor cell suspension. Macrophages were characterized based on expression of macrophage markers F4/80 and CD11b. Double-positive cells were further sorted for downstream RNA analysis (Figure S3A). Splenic macrophages were also isolated because they are resident tissue macrophages known to be abundantly present in spleen tissue. To test if our methodology could be successfully applied on fluorescence-activated sorted cells, we performed qRT-PCR analysis of both tumor-associated macrophages and splenic macrophages by the Microscale gene expression analysis method. We wanted to develop a method to perform gene expression analysis from a large number of FACS sorted cell samples at the same time, and so therefore we sorted the cells in PBS and stored them in a −80 refrigerator for later analysis. *Actb* gene expression was detected in both tumor and splenic macrophages, but Ct values were very high and considerable variation was observed in the technical replicates from the sorted cells (Fig. 5A). High Ct values can be a result of gene analysis from degraded RNA samples, and earlier reports also show that storage of RNA in PBS leads to its degradation, with biased amplification of different parts of the transcripts leading to an increase in Ct values for the target genes^27^. These results are also in line with previous results and they additionally suggest that even storage of sorted RNA at a lower temperature could not prevent RNA degradation. Therefore, we next isolated 5,000 tumor-associated macrophages directly into the extraction buffer supplied in the PicoPure RNA Isolation kit and immediately extracted RNA by completing the isolation procedure. The qRT-PCR analysis of fresh FACS-sorted cells show relatively lower Ct values compared to stored FACS-sorted macrophages, but showed higher values than cultured macrophages. The Ct values of *Actb* from freshly isolated FACS-sorted macrophages showed no significant variation between replicates (Fig. 5A). Further, we checked the expression of *iNOS* (M1 marker) and *Arg1* (M2 marker) to check for macrophage polarization. A very low variation between Ct values of replicates for *Nos2* (*iNOS*) and *Arg1* was observed in the murine BMDMs and for fresh FACS-sorted macrophages; however, a notable variation was seen in stored FACS-sorted macrophages (Fig. 5B and C). In tumor-associated macrophages, a high expression of *Arg1* was observed, indicating that the macrophages found in tumors are M2 macrophages. These findings are consistent with earlier data from studies of pancreatic cancers, wherein M2 macrophages were shown to promote tumor progression^28^. The expression of *Nos2* was not detected in stored FACS-sorted macrophages, but a relatively higher expression was seen in fresh FACS-sorted macrophages (Fig. 5C). Further, we investigated the reproducibility of gene expression data for *Actb* gene from fresh FACS-sorted macrophages in three different biological replicates. Ct values were almost the same within the three biological replicates, showing significant differences between (+) RT and (−) RT PCR reactions (Figure S3B). Our data suggests that performing gene expression analysis from 5,000 freshly sorted cells in an extraction buffer will result in the generation of efficient and reproducible gene expression data.

Several kits are also available commercially that claim to repair degraded RNA to enable qRT-PCR analysis out of poor quality RNA samples. Since the RNA from FACS-sorted stored macrophages had degraded RNA, we attempted to synthesize cDNA by using Thermo SSIV kit which claims to reduce Ct value by 8 Ct values due to highly efficient cDNA synthesis process. The cDNA synthesized in this manner was compared with cDNA synthesized from SuperScript Verso kit and probed for expression of the *Actb* gene in splenic and tumor-associated macrophages. Our analysis showed no significant improvement in detection of Ct values and a high variation among replicates was observed (Figure S3C). We recommend performing RNA isolation immediately from FACS-sorted cells in extraction buffer without any intermediate storage step to achieve efficient RNA recovery for downstream qRT-PCR analysis.

## Discussion

Biological tissues consist of a heterogeneous population of various cellular subtypes that differentially respond to changes in the microenvironment by transcriptional modulation. Different cellular subpopulations in the same tissue are known to trigger differential activation of multiple cellular signaling cascades and result in altered functional outputs^29–31^. In complex diseases like cancer, cross-talk of multiple cellular subtypes leads to the formation, progression, and maintenance of tumors^32,33^. Recent literature also suggests that not only tumor cells, but stromal and immune cells also modulate the plasticity of tumor phenotype. Transcriptional regulation changes in these non-tumor cells have a profound impact on the formation and progression of tumors^34,35^. Because immune cells such as macrophages are present in very low amount in tissues and tumors, it becomes important to be able to isolate sufficient RNA from small number of cells to study transcriptional adaptations.

In this study, we standardized our method by comparing different numbers of cultured and primary macrophage cells to determine the minimum number of cells necessary to conduct efficient and reproducible gene expression analysis. We found that a minimum of 5,000 cells are sufficient to study transcriptional changes using our methodology. Nevertheless, we could detect gene expression from as low as 500 cells, but even so, a very high variation was observed in Ct values between replicates. Moreover, if transcript expression is studied from a lower number of cells, fewer transcripts were detected as opposed to analysis of 5,000 cells using our method, and a large number of cells using the TRIzol method. The RNA isolated from 5,000 cells is able to generate ample cDNA and can be used to perform qRT-PCR analysis for at least 200 genes. Gene expression studies have also recently been conducted from flow-sorted single cells; however, such methods can introduce biases due to additional amplification steps^29^. Another commonly used approach to overcome limitations in biological or clinical samples is to pool samples that result in a loss of inter-individual variation in *in vivo* studies^19^. Our methodology enables accurate detection of relative transcript levels without introducing any significant bias.

Subtype-specific surface marker macrophage antibodies are widely used to distinguish and sort the subpopulation of macrophages from biological tissues that can be further used for downstream analysis such as transcriptomics. We analyzed gene expression from 5,000 FACS-sorted macrophages from mice pancreatic tumor tissue. We could detect Ct values in the range of 20–25 for *Actb* genes in these samples. Further, we could establish that the macrophages that infiltrate pancreatic tumors from orthotropic mice model are M2 macrophages, as reported earlier in the literature. However, RNA extraction should be performed immediately after FACS-sorting of cells because storage of cells in buffer leads to degradation of RNA and consequently shows high Ct values. We were not able to achieve optimal or reproducible detection of transcripts from stored FACS-sorted samples, even after attempting multiple kits supposed to recover good quality cDNA yields from degraded RNA samples. Taken together, our strategy can be used to study transcript expression from isolated cell populations of biological tissues, provided that FACS sorting and RNA extraction are processed simultaneously.

In this study, we have developed a cost-effective, rapid, and highly specific method for studying quantitative gene expression changes from a very small number of cultured or FACS-sorted macrophages isolated from tissues. The transcriptional changes examined by our method show similar-fold change regulations compared to RNA isolated from a large number of cells by the commonly used TRIzol method. We further demonstrated that this method is also applicable to macrophages isolated from pancreatic tumors and other tissues. To our knowledge, this is the first study that describes gene expression analysis from as low as 5,000 macrophages without using an amplification step that might introduce any bias to the detection of gene expression values. Although this Microscale gene expression method has been standardized for macrophage cells, it can be effectively applied to a wide array of cellular subtypes found in very small numbers in different types of biological tissues. Thus, our method provides a cost-effective approach for understanding quantitative low-throughput transcriptional changes in many genes, without introducing noise that can arise when pooling biological samples.

## Materials and Methods

### Chemicals and Kits

The cell culture reagents were purchased from Gibco, Invitrogen (USA). The Live/Dead Aqua staining kit was purchased from Invitrogen. Anti-F4/80-PE and Anti-CD11b-Pacific Blue Abs were purchased from eBiosciences (USA). ARCTURUS PicoPure RNA Isolation Kit (Catalog No.-KIT0204), Verso cDNA Synthesis Kit (Catalog No.-AB-1453/B), SuperScript VILO MasterMix (Catalog No.-11755050) and SuperScript IV First Strand Synthesis System (Catalog No.-18091050) were purchased from ThermoFisher Scientific (USA). The SYBR Green MasterMixes-FastStart Universal SYBR Green Master (Rox) (Catalog No.-04913914001), PowerUP SYBR Green MasterMix (Catalog No.-A25742) and Sso Advanced Universal IT SYBR Green SuperMix (Catalog No.-1725270) were purchased from Roche (Lifescience), Applied Biosystems (ThermoFisher Scientific) and Bio-Rad respectively.

### Culturing of U937 Cells, KPC cells, and Murine Bone Marrow-Derived Macrophages

Monocytic U937 (ATCC) cell line was cultured in Dulbecco’s Modified Eagle’s Medium (DMEM), with 10% FBS, 1 mM glutamine, 50 U/ml penicillin and 50 μg/ml streptomycin.

KPC-k1 cells are a murine cancer cell line derived from a pancreatic tumor formed in the congenic KPC spontaneous progression mouse model. These cells were cultured in Dulbecco’s Modified Eagle’s Medium (DMEM), with 10% FBS, 1 mM glutamine, 50 U/ml penicillin and 50 μg/ml streptomycin.

Bone marrow-derived macrophages (BMDMs) were isolated from C57BL/6 mice as reported earlier^36^. The protocol for *ex vivo* BMDM cultures was approved by University of Nebraska Medical Center institutional animal care and use committee (IACUC). Briefly, BMDMs were isolated from 5 to 6-week-old C57BL/6 mice. Femur and tibia were isolated. Epiphysis of the bones was cut, and the marrow cavity was flushed out with α-MEM medium from one end of the bone using a sterile 21-gauge needle to take out the cells from marrow cavity. The cells were passed through a syringe with 21-gauge needle to make a single cell suspension. The cell suspension was layered on a Ficoll-Hypaque gradient in a ratio of (1:4) to collect hematopoietic stem cells – the precursor of macrophages. The cells were finally cultured in DMEM media supplemented with 10% FBS, 20% L-929-cell conditioned media, 100 U/ml penicillin, 100 µg/ml streptomycin, and 2 mM L-glutamine for next seven days to differentiate into macrophages.

### RNA Isolation by TRIzol Method and Kit-based Method

RNA isolation from a large number of cells: RNA was isolated from 2 million U937 cells or 1 million murine BMDMs using TRIzol reagent (Invitrogen) according to the manufacturer’s instructions. RNA was isolated using chloroform-isopropanol method as reported earlier^37^.

RNA isolation from a small number of cells: The U937 cells or primary bone marrow macrophages were counted using trypan blue dye. The RNA from fixed number of cells was isolated using Picopure RNA isolation kit according to the manufacturer’s instructions. The fixed numbers of cells as indicated in the experiment were pelleted and resuspended in 100 µl extraction buffer and incubated at 42 °C for 30 minutes (min). During incubation, the column was pre-conditioned using 250 µl of conditioning buffer by centrifuging at 16,000 g for 1 min. After 30 min of incubation, 100 µl of 70% ethanol was mixed with the lysate. The combined lysate mix was loaded onto the column and washed with wash buffer1 (once) and wash buffer 2 (twice) by centrifugation. A dry spin was given next to remove the traces of ethanol. The RNA was finally eluted in 20 µl of elution buffer and stored at −80 °C for further use.

### Orthotropic Mice Model of Pancreatic Cancer for Isolation of Macrophages from Tumor

C57BL/6 albino mice were used for the study. Mice were housed as per the guidelines of our institutional animal care and use committee (IACUC). Syngeneic KPC-k1 cells (5 × 10^4^) were orthotopically implanted in the pancreas of 6–8-week-old female C57BL/6 albino mice. Tumor volume was regularly monitored with the help of caliper. When tumor size reached 1cc, mice were sacrificed, and tumor tissue and spleen were collected for further FACS sorting analysis. Animal protocols were in accordance with the NIH Guide for the Care and Use of Laboratory Animals and were approved by the University of Nebraska Medical Center Animal Care and Use Committee.

### FACS sorting of Macrophages from Spleen and Tumors

Single cell suspensions were made from pancreatic tumor and spleen samples. The cells were stained with Live/Dead Aqua stain for 15 min. After staining the cells were washed with PBS. The cells were again stained with anti-F4/80-PE and anti-CD11b-Pacific Blue for 20 min. After staining, the cells were washed with PBS and fixed with 4% paraformaldehyde. The cells were washed and finally resuspended in PBS. The cells were acquired and sorted through BD FACSAriaII sorter in class 2A biosafety hood.

### cDNA Synthesis and Real Time PCR

1 µg of RNA isolated using TRIzol method was reverse transcribed to complementary DNA (cDNA) using Verso cDNA synthesis kit according to the manufacturer’s instructions. Briefly, a 20 µl reaction was setup by adding 4 µl of cDNA synthesis buffer, 2 µl dNTP mix, 1 µl of random hexamer primers, 1 µg RNA, 1 µl RT enhancer and 1 µl of Verso Enzyme Mix. The synthesis was carried out at 42 °C for 30 min followed by inactivation at 95 °C for 2 min. The synthesized cDNA was diluted 20 times, and 3 µl cDNA was analyzed in a 10 µl reaction mix consisting of 1X SYBR dye (Roche), forward and reverse primers. The real-time PCR primers used in the study are experimentally validated sequences taken from Primer Bank database (https://pga.mgh.harvard.edu/primerbank/). The list of primers used in this study has been shown in Table S1. Quantitative real-time PCR was performed using QuantStudio5 Real-Time PCR system (Thermo Fisher Scientific).

10 µl of RNA isolated using Picopure RNA isolation method was reverse transcribed by using SuperScript VILO MasterMix Synthesis Kit according to the manufacturer’s instructions. Briefly, 10 µl RNA was mixed with 4 µl of SuperScript VILO Mastermix and 6 µl nuclease free water. The synthesis was carried out at 25 °C for 10 min, 42 °C for 60 min followed by termination at 85 °C for 5 min. The cDNA synthesis using SuperScript IV First Strand Synthesis System was carried out by adding 1 µl of random hexamers and 1 µl dNTP mix to 11 µl of RNA. The mix was incubated at 65 °C for 5 min. To the mix, 4 µl SSIV buffer, 1 µl DTT, 1 µl Ribonuclease inhibitor and 1 µl of SSIV reverse transcriptase enzyme was added. The synthesis was carried out at 23 °C for 10 min, 55 °C for 10 min, followed by inactivation at 80 °C for 10 min. The synthesized cDNA was diluted 20 times and 3 µl cDNA was analyzed in a 10 µl reaction mix consisting of 1X SYBR dye, forward and reverse primers. All the three SYBR dyes were used at 1X concentration in qPCR mix and threshold cycles (Ct) were detected using QuantStudio5 Real-Time PCR system. The cycling parameters used for AB PowerUp SYBR Bio-Rad and Roche SYBR was: pre-incubation at 50 °C for 2 min followed by 95 °C for 2 min, 45 cycles of 95 °C for 15 sec, 60 °C for 1 min. The cycling parameter used for Bio-Rad and Roche SYBR was: pre-incubation at 98 °C for 2 min, 45 cycles of 95 °C for 15 sec, 60 °C for 1 min. The specificity of all the reactions was determined by running a melting curve analysis at the end of the PCR cycles. The steady state expression of various genes was normalized to the β-actin gene wherever indicated.

### Statistical Analysis

The statistical significance was determined by unpaired student’s t-test (two-tailed, unpaired) or one-way ANOVA test (Bonferroni’s multiple comparisons) using GraphPad Prism, GraphPad Software Inc., San Diego, USA. ***Denotes p-value < 0.001, **Denotes p-value < 0.01, *Denotes p-value < 0.05. p-value < 0.05 was considered significant and ns stands for non-significant.

## Electronic supplementary material

Supplementary Figures S1-S3 and Supplementary Table 1
