# Supplementary material for: Microscale Gene Expression Analysis of Tumor-Associated Macrophages
Source: Sci Rep. 2018 Feb 5;8:2408. doi: 10.1038/s41598-018-20820-4 (PMC5799305; doi:10.1038/s41598-018-20820-4)
Supplement: Supplementary file 1 — Supplementary Figures S1-S3 and Supplementary Table 1 [file 41598_2018_20820_MOESM1_ESM.pdf]

Microscale Gene Expression Method for Analysis of Tumor-Associated Macrophages

Kuldeep S. Attri[1], Kamiya Mehla[1], Surendra K. Shukla[1], and Pankaj K. Singh[1,2, 3,4,*]

[1] The Eppley Institute for Research in Cancer and Allied Diseases, University of Nebraska Medical Center, Omaha, Nebraska, USA.

[2] Department of Biochemistry and Molecular Biology, University of Nebraska Medical Center, Omaha, Nebraska, USA.

[3] Department of Pathology and Microbiology, University of Nebraska Medical Center, Omaha, Nebraska, USA.

[4] Department of Genetics Cell Biology and Anatomy, University of Nebraska Medical Center, Omaha, Nebraska, USA.

<u>Supplementary Information:</u>

Supplementary Figure Legends:

Supplementary Figures:

Supplementary Table:

Supplementary Figure Legends:

Figure S1. Detection of genomic DNA contamination in RNA isolated from a lower number of U937 cells.

The graph shows Ct values of (A) *18SrRNA*, (B) *ACTB*, and (C) *GAPDH* in (+) RT and (-) RT reactions probed at 1:20 dilution of cDNA. The data is represented as mean ± SEM from n = 3. The unpaired two-tailed student's t-test was utilized for comparison between groups. *** denotes p-value < 0.001, ** denotes p-value < 0.01, * denotes p-value < 0.05 and ns stands for non-significant.

Figure S2. Reproducibility of gene expression analysis method from a low number of macrophages.

The graph represents variability in expression of (A) *18SrRNA*, (B) *ACTB* and (C) *GAPDH* genes from three different experiments. The RNA isolated from 5,000 and 1,000 U937 macrophages was probed at cDNA dilution of 1:20. The data is represented as mean ± SEM from n = 3. The significant differences in mean Ct values between biological replicates were determined by one-way ANOVA. *** denotes p-value < 0.001, ** denotes p-value < 0.01, * denotes p-value < 0.05 and ns stands for non-significant.

Figure S3. Expression analysis of genes from fresh and stored FACS-sorted macrophages.

(A) Representative scatter plot for FACS-based characterization of tumor isolated macrophages. (B) The graph shows Ct values of *Actb* in (+) RT and (-) RT reactions probed at 1:20 dilution of cDNA synthesized from freshly isolated tumor macrophages by Microscale gene expression analysis method. (C) The graph shows relative expression of *Actb* gene from stored FACS-sorted splenic and tumor macrophages by synthesizing cDNA using Verso cDNA synthesis kit and Superscript IV First Strand Synthesis System. The data is represented as mean ± SEM from n = 3. The unpaired two-tailed student's t-test was utilized for comparison between groups. *** denotes p-value < 0.001, ** denotes p-value < 0.01, * denotes p-value < 0.05 and ns stands for non-significant.

Supplementary Figures:

Figure S1

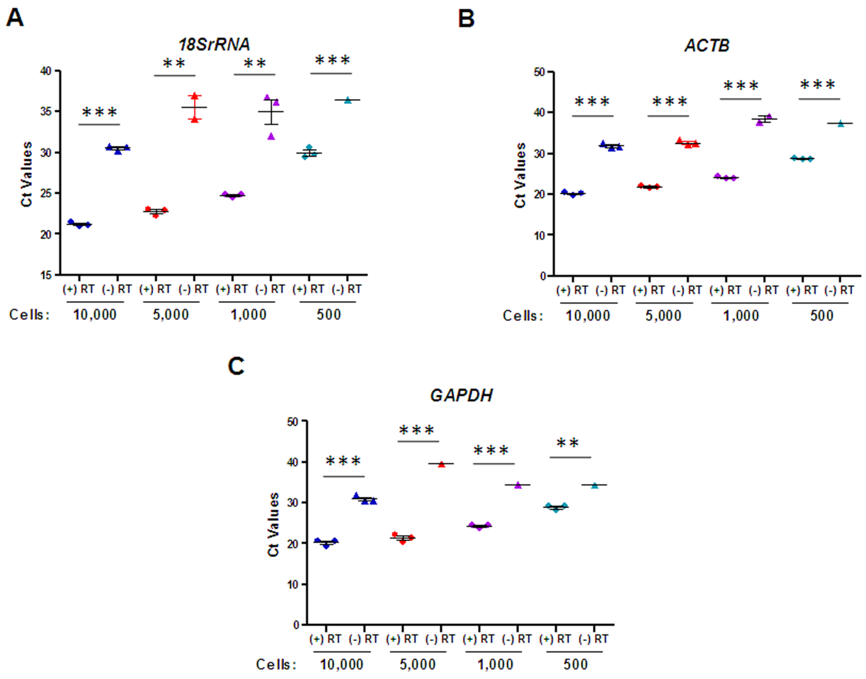

Figure S2

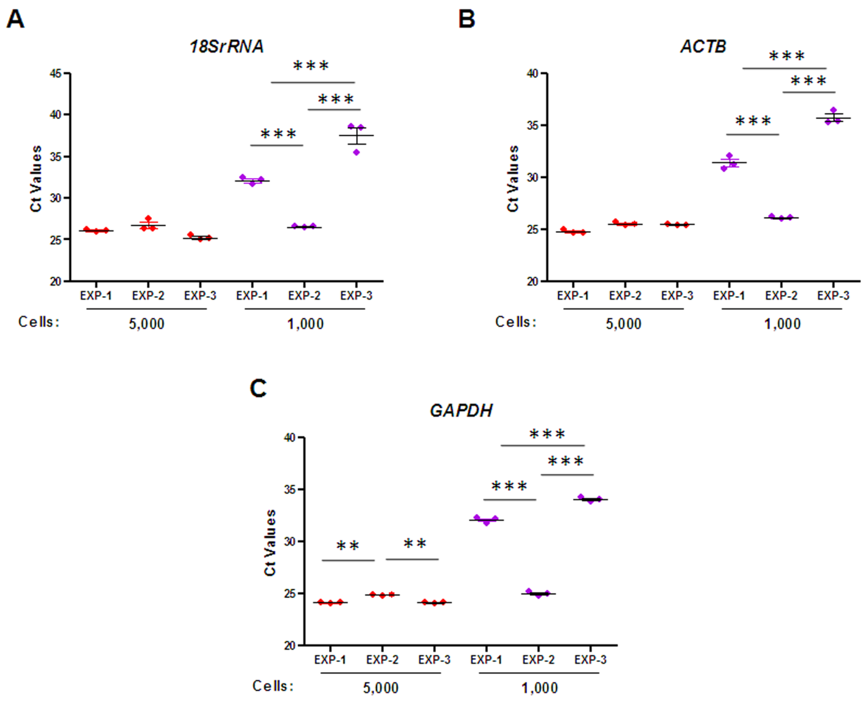
A
18SrRNA
Ct Values
45
40
35
30
25
20
EXP-1
EXP-2
EXP-3
EXP-1
EXP-2
EXP-3
Cells:
5,000
1,000
***
***
***
B
ACTB
Ct Values
40
35
30
25
20
EXP-1
EXP-2
EXP-3
EXP-1
EXP-2
EXP-3
Cells:
5,000
1,000
***
***
***
C
GAPDH
Ct Values
40
35
30
25
20
**
**
***
***
***
EXP-1
EXP-2
EXP-3
EXP-1
EXP-2
EXP-3
Cells:
5,000
1,000

Figure S3

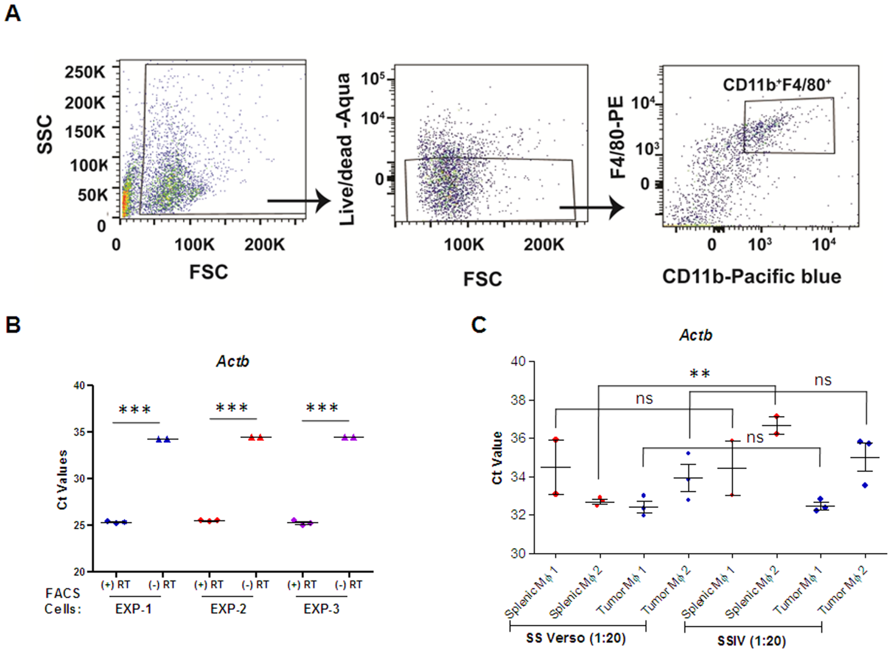
A
SSC
FSC
Live/dead -Aqua
FSC
F4/80-PE
CD11b-Pacific blue
CD11b⁺F4/80⁺
B
Actb
Ct Values
FACS Cells:
(+) RT
(-) RT
EXP-1
EXP-2
EXP-3
C
Actb
Ct Value
ns
**
ns
ns
Splenic Mϕ 1
Splenic Mϕ 2
Tumor Mϕ 1
Tumor Mϕ 2
SS Verso (1:20)
SSIV (1:20)

Supplementary Table:

Table 1: List of primers used in this study.

S. No.	Gene	Organism	Forward Primer (5' – 3')	Reverse Primer (5' – 3')
1	*ACTB*	HUMAN	GTCCACCGCAAATGCTTCTA	TGCTGTCACCTTCACCGTTC
2	*GAPDH*	HUMAN	ATGGGGAAGGTGAAGGTCG	GGGGTCATTGATGGCAACAATA
3	*18SrRNA*	HUMAN	GCTTAATTTGACTCAACACGGGA	AGCTATCAATCTGTCAATCCTGTC
4	*MCP-1*	HUMAN	GCTCATAGCAGCCACCTTCATTC	TGCAGATTCTTGGGTTGTGGAG
5	*MCP-3*	HUMAN	CCCAGGGGCTTGCTCAG	CATGGCTTGTTTTCAGTTCAGTCA
6	*TNFA*	HUMAN	GCCTGTAGCCCATGTTGTAG	TTGGGAAGGTTGGATGTTCG
7	*HIF1A*	HUMAN	CGTTCCTTCGATCAGTTGTC	TCAGTGGTGGCAGTGGTAGT
8	*HIF2A*	HUMAN	GCGCTAGACTCCGAGAACAT	TGGCCACTTACTACCTGACCCTT
9	*TPI1*	HUMAN	AAGTTCTTCGTTGGGGGAAAC	GTAGGGGGAGCACAAACCAC
10	*SDHA*	HUMAN	CAAACAGGAACCCGAGGTTTT	GCCTCAGAAAGGCCAAATGC
11	*Actb*	MOUSE	GGCTGTATTCCCCTCCATCG	CCAGTTGGTAACAATGCCATGT
12	*Gapdh*	MOUSE	AGGTCGGTGTGAACGGATTTG	TGTAGACCATGTAGTTGAGGTCA
13	*Il1b*	MOUSE	AAGGGCTGCTTCCAAACCTTTGAC	ATACTGCCTGCCTGAAGCTCTAGT
14	*Il12b*	MOUSE	GGAAGCACGGCAGCAGAATA	AACTTGAGGGAGAAGTAGGAATGG
15	*Nos2*	MOUSE	GTTCTCAGCCCAACAATACAAGA	GTGGACGGGTCGATGTCAC
16	*Ym1*	MOUSE	GCCACTGAGGTCTGGGATGC	TCCTTGAGCCACTGAGCCTTC
17	*Vegfa*	MOUSE	CCACGACAGAAGGAGAGCAGAAGTCC	CGTTACAGCAGCCTGCACAGCG
18	*Rpl13a*	MOUSE	GAGGTCGGGTGGAAGTACCA	TGCATCTTGGCCTTTTCCTT
19	*Pkm1*	MOUSE	ATTACCAGCGACCCCACAG	TAGAAGAGGGGCTCCAGAGG
20	*Pkm2*	MOUSE	AGGATGCCGTGCTGAATG	TAGAAGAGGGGCTCCAGAGG
21	*Otc*	MOUSE	GCTAGCAGAGCAGTATGCCA	ATACATTGCCTCCACGTGCT
22	*Mrc1*	MOUSE	AAGGCTATCCTGGTGGAAGAA	AGGGAAGGGTCAGTCTGTGTT
23	*MhcII*	MOUSE	TTTGCTTTCTGAAGGGGGCA	TCGCCCATGAACTGGTACAC
24	*Mgl1*	MOUSE	CAGAATCGCTTAGCCAATGTGG	TCCCAGTCCGTGTCCGAAC
25	*Mgl2*	MOUSE	TTCAAGAATTGGAGGCCACT	CAGACATCGTCATTCCAACG
26	*Hif1a*	MOUSE	AATACATTTTCTCTGCCAGTTTTCTG	TTGCTGCATCTCTAGACTTTTCTTTT
27	*Gpt*	MOUSE	CTCTAAGGGCTACATGGGCG	ACCTGCTCCGTGAGTTTAGC
28	*Glul*	MOUSE	CCTTCCGCAAAGACCCCA	CATTCCAAACCAGGGGTGCT
29	*Fizz1*	MOUSE	CCTGCTGGGATGACTGCTA	TGGGTTCTCCACCTCTTCAT
30	*Cd11c*	MOUSE	GGATAGCCTTTCTTCTGCTGT	TGTAGAGGCCACCTATTTGGTT
31	*Ass*	MOUSE	GCCAAGTGTACATCCTCGGT	GACCTTGCTCTGAAGGCGAT
32	*Asl*	MOUSE	GGGAAGCTACACACAGGACG	GCTGAGCTCTCTGCAAGTGT
33	*Arg1*	MOUSE	CCACAGTCTGGCAGTTGGAAG	GGTTGTCAGGGGAGTGTTGATG
